# Barriers to access prevention of mother-to-child transmission for HIV positive women in a well-resourced setting in Vietnam

**DOI:** 10.1186/1742-6405-5-7

**Published:** 2008-04-17

**Authors:** Thu Anh Nguyen, Pauline Oosterhoff, Yen Pham Ngoc, Pamela Wright, Anita Hardon

**Affiliations:** 1 Faculty of Public Health, Hanoi Medical University, Hanoi, Vietnam; 2 Medical Committee Netherlands Vietnam, Hanoi, Vietnam; 3 Amsterdam School of Social Science Research, University of Amsterdam, Amsterdam, The Netherlands

## Abstract

**Background:**

According to Vietnamese policy, HIV-infected women should have access at least to HIV testing and Nevirapine prophylaxis, or where available, to adequate counselling, HIV infection staging, ARV prophylaxis, and infant formula. Many studies in high HIV prevalence settings have reported low coverage of PMTCT services, but there have been few reports from low HIV prevalence settings, such as Asian countries. We investigated the access of HIV-infected pregnant women to PMTCT services in the well-resourced setting of the capital city, Hanoi.

**Methods:**

Fifty-two HIV positive women enrolled in a self-help group in Hanoi were consulted, through in-depth interviews and bi-weekly meetings, about their experiences in accessing PMTCT services.

**Results:**

Only 44% and 20% of the women had received minimal and comprehensive PMTCT services, respectively. Nine women did not receive any services. Twenty-two women received no counselling. The women reported being limited by lack of knowledge and information due to poor counselling, gaps in PMTCT services, and fear of stigma and discrimination. HIV testing was done too late for optimal interventions and poor quality of care by health staff was frequently mentioned.

**Conclusion:**

In a setting where PMTCT is available, HIV-infected women and children did not receive adequate care because of barriers to accessing those services. The results suggest key improvements would be improving quality of counselling and making PMTCT guidelines available to health services. Women should receive early HIV testing with adequate counselling, safe care and prophylaxis in a positive atmosphere towards HIV-infected women.

## Introduction

Prevention of mother-to-child-transmission (PMTCT) of an HIV infection is a politically and scientifically accepted approach to reduce the impact of HIV, especially on the children. Early in the 90's, prophylaxis by Zidovudine during pregnancy was found to be effective for PMTCT [1,2]. Later, WHO introduced several simplified anti-retroviral (ARV) prophylaxis regimens [3]. However, experiences in many countries suggested that ARV prophylaxis for PMTCT alone had only limited impact. Even in facilities where ARV prophylaxis was available, still a number of pregnant women would drop out at different steps of the health care process [4]. Many countries reported low uptake of HIV testing. The most important barrier to use the services was found to be fear of stigma and discrimination among HIV positive pregnant women [5-8]. Poor counselling or lack of counselling meant that HIV positive pregnant women lacked awareness on PMTCT opportunities, which limited their access to these services [9,10]. Worryingly some studies revealed that health staff were unwilling to provide appropriate care for HIV positive pregnant women, often because of their own fear or lack of knowledge [11-13].

To optimize the effectiveness of PMTCT, WHO promotes a four-pronged comprehensive approach, aimed at improving maternal and child health in the context of an HIV epidemic. This approach promotes routine HIV testing and counselling for pregnant women. If a woman found to be HIV positive wants to continue her pregnancy, she should receive clinical management and HAART for herself, if eligible, or at least ARV prophylaxis. For those who want to terminate their pregnancy, safe abortion should be offered where available. Pregnant women should also receive counselling on safe infant feeding choices and appropriately referred for continued care for themselves and their children after delivery [3].

Results of many studies in high HIV prevalence settings, such as sub-Saharan Africa, suggested that PMTCT coverage was low, and explored the gaps at each stage of the PMTCT cascade of services. There have been very few studies on PMTCT Asian countries where HIV prevalence is still low. Particularly rare are reports based on the real experience of HIV-infected women trying to access PMTCT services.

In Vietnam, HIV prevalence studies confirm increasing HIV infection rates in high-risk populations, as well as increasing spread from them to the general population. The first HIV pregnant Vietnamese women were identified in 1993. The HIV prevalence among pregnant women has since increased from 0.03% in 1994 to 0.38% in 2006 [14]. Of the 1.8 – 2 million women who give birth annually, an estimated 3000 HIV positive women delivered in 2000 [15], 6000 in 2002 [16], and 6,500 – 8,000 in 2005 [17].

Operational guidelines on PMTCT in Vietnam are not yet available, but according to the national policy, HIV testing should be offered to all pregnant women delivering at state facilities. Since 2001, state health facilities are required by policy and law to provide prophylactic single-dose Nevirapine (SD-NVP) free of charge for all HIV positive pregnant women [18-20]. These services are, however, not available everywhere, partly because of the weakness of the health system in general, especially in the provinces. In the big cities where internationally-funded projects support the PMTCT program, the availability of ARV combination prophylaxis and free infant formula should make it possible for the program to work more effectively [11]. However, an effective program requires strong collaboration between the different services, including antenatal care (ANC), obstetrical care, anti-retroviral therapy (ART) programs, voluntary counselling and testing (VCT), abortion and family planning (FP).

Even when the simplest PMTCT program was applied, with only HIV testing and SD-NVP prophylaxis, still the number of women receiving PMTCT in a given year in Vietnam has been consistently lower than the estimated number of HIV positive women expected to deliver. Among the HIV positive women who were detected, as few as 25% received prophylactic SD-NVP [16]. In the rural areas, the health services are not yet strong enough to deliver adequate PMTCT services. But even in the best funded and equipped urban settings, women have to find their way through a maze of fragmented services, with the result that many women who should be getting PMTCT are not. We have described the antenatal care and testing services used by 670 women in Hanoi, not related to HIV infection. There was a lack of choices for pregnant women to enter PMTCT programs, mainly because of late offering of HIV testing and inappropriate counselling about possible PMTCT interventions [Thu Anh N, et al., submitted for publication].

The present study focused on the experiences of 52 HIV-infected women looking for assistance in a relatively well-serviced area in the capital city, Hanoi. They were pregnant and should have received PMTCT advice and services. Specifically, we investigated:

(1) how many women received minimal, comprehensive, and optional PMTCT services? and

(2) what were their experiences in accessing PMTCT services in a well-resourced urban setting?

with the aim of providing indications on how to improve the services and reduce the risk of HIV transmission from mother to child in Vietnam.

## Methods

Minimal PMTCT service is defined as access to HIV testing and at least SD-NVP for mother at delivery and NVP for the child post-delivery, while comprehensive PMTCT would include testing with counselling, access to HIV infection staging for treatment, ARV prophylaxis for mothers and exposed children, and infant formula. Abortion and Caesarean section are considered optional services for PMTCT.

Hanoi was selected as study site because comprehensive PMTCT care is theoretically available there. Hanoi is the capital of Vietnam, with an estimated 3.2 million inhabitants in 14 districts (9 urban and 5 suburban). The HIV epidemic in Hanoi has been increasing rapidly in size since 1994. HIV infection is predominantly concentrated among injecting drug users, but increasingly among female sex workers, and is starting to spread to the general population. In the year 2006, HIV prevalence among pregnant women attending ANC clinics in the Hanoi was found to be 0.38% [14].

In Hanoi, HIV testing and counselling is routinely carried out for pregnant women who deliver in all obstetric hospitals/clinics from the district to national level, where more than 85% of pregnant women choose to deliver. Because HIV prevalence in Hanoi is low, the health system cannot provide ARV prophylaxis in all facilities, but only in referred hospitals at provincial and national level. If a pregnant woman in Hanoi is found to be HIV positive, she should be referred to the appropriate hospital to get the care she needs, even if her chosen ANC site cannot provide all services itself.

HIV-infected women are extremely marginalized in society as HIV is highly stigmatized in Vietnam, making it very difficult for researchers to contact HIV-infected pregnant women. Since April 2004, our research team has worked with mass organizations and hospitals, setting up a referral system for women eligible for a self-help group for HIV-infected mothers in Hanoi, called the Sunflower group. The Sunflower group members deposited the group's posters, leaflets, and name cards in 26 health facilities at all levels in Hanoi. At each facility, IEC materials were posted in waiting places, testing sites, and examination wards. Core members visited obstetric hospitals, general hospitals, paediatric hospitals, and VCT sites to make informal contact with potential members and to refer them to the group. HIV-infected pregnant women were referred to the group by hospital staff or by core members of the self-help group. In the self-help group, the women received care and support for themselves and their families. The researcher participated as co-facilitator in workshops on creative communication aimed at helping the women to communicate better about the many problems they experience in relation to their HIV infections. During the workshop, the researcher observed and collected their concerns through both oral and physical expressions and stories. These workshops also helped the researcher to gain trust from women whose stories constitute the data of the study.

Fifty-two HIV-infected women agreed to participate in the study and to share their stories through in-depth interviews. Inclusion criteria were women who found out that they were HIV positive before or during pregnancy and had completed the pregnancy. The women were enrolled in the study at different stages of pregnancy, between 12 weeks and 40 weeks. Basic information on their characteristics was collected when the women entered the cohort. They were interviewed for on average two hours about their ANC seeking behaviours in relation to PMTCT and about their use of and access to PMTCT services including: HIV testing and counselling, ARV prophylaxis for them and their children, and replacement feeding. Retrospective data was collected not on only one occasion but through individual in-depth interviews, bi-weekly meetings with the group, household visits, and counselling via a telephone hotline.

The interviewers were four trained public health and social science researchers. Institutional ethical approval was obtained from the Scientific Committee of Hanoi Medical University and written informed consent was obtained from all interviewees. The interviews were conducted privately and anonymously. A code book was developed focusing on key findings and terminologies. The transcripts of the semi-structured interviews were coded, entered and analyzed using N-VIVO software.

At the time the respondents entered the group, their ages were between 18 and 36 years. The youngest child was less than 1 year old. Two HIV infected women desired to have a child, although they knew their positive status when they got pregnant. The majority of the women (49/52) reported that they had been infected by their husband and the remaining three were infected through sexual contact with a casual partner. Ten of them had graduated from college or university, two had finished primary school, and the rest had completed secondary and high schools. The majority was married and worked in the informal sector. Only nine had health insurance. The background information on the respondents' use of ANC and PMTCT services is presented in Table 1.

**Table 1:** Access to ANC, delivery care and PMTCT among 52 HIV positive pregnant women

Service	Number of respondents	
ANC, number of visits		
1–2		8
3		9
4–9		17
> 9		18

Facility attended	For ANC	For delivery

National hospital	16	26
Provincial/sector hospital	17	18
District hospital	11	4
Commune health station	12	4
Private clinic	11	0
Pre-test counselling		
Yes		15
No		35
HIV tested at		
Before pregnancy		2
< 23rd week		8
23–36^th ^week		25
> 36th week		2
Labour		15
Post-test counselling		
Yes		27
No		25
ARV prophylaxis for mother		
SD NVP		27
ARV combination prophylaxis		4
None		21
Abortion		0
Delivery method		
Vaginal delivery		42
C-section		9
Forceps		1
Number of children delivered		52
ARV prophylaxis for child		
NVP		26
None		26
Free infant formula		
Received		26
Not received		26

## Results

### 1. Access to minimal services for PMTCT

**Figure 1:**
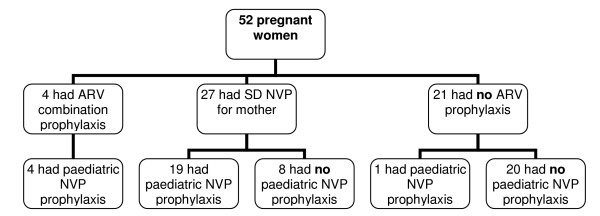
Use of minimal service for PMTCT among HIV positive women in Hanoi.

To reduce the impact of HIV infection on pregnant women and their babies, women who are HIV positive require a minimal type of care before, during and after delivery, which includes HIV testing and ARV prophylaxis for both mother and infant. All of the study population had tested positive for HIV at some time, and therefore should have received at least SD-NVP for the mother and NVP prophylaxis for the newborn, to reduce the risk of transmission to the child. As the flow chart in Figure 1 shows, among the 52 women, only 23 (44%) mother-child pairs received ARV prophylaxis, while 20 pairs did not receive any prophylaxis at all (Figure 1).

Also, among the 35 women who were tested before the 36^th ^week of gestation, and could have received ARV from that time, 17 women did not have any ARV prophylaxis at all and 14 were only given the treatment at the time of labour (Table 2).

**Table 2:** ARV prophylaxis provided according to time of HIV testing

ARV regimen	Tested at 36^th ^week or earlier	Tested after 36^th ^week
ARV combination	4	0
SD NVP	14	13
No ARV prophylaxis	17	4

Total	35	17

One reason for this disappointing record was that in many health care facilities, the ARV was not consistently available. Often even single dose NVP for women who were tested only at the time of labour was lacking. Even in the two PMTCT sites in the city, stock-out of ARV every few weeks was observed.

Another reason was that the women received no counselling, or counselling that lacked information about PMTCT and the options for women to receive PMTCT. Among the 52 women, there were 15 who either lacked knowledge about the infection and testing (8) or had never thought about their own risk of infection (7). Most of the women were not aware that medication could prevent MTCT.

Many HIV infected mothers reported that they did not receive treatment for their babies. The doctors explained to them that NVP was provided to the hospital as a large bottle (200 ml) of syrup. Once it was opened, it could not be kept for long, but very few HIV exposed children were identified each day. That means that each bottle was not fully used, and that later, drugs were lacking when supplies ran out. Another problem was that although the drug should be given to the baby for seven days, the mother often leaves the hospital before that time is up. Commonly the syrup is given to the mother to take home in a syringe, but that is an inconvenient way to transport a syrup and it often gets lost before use, so that mothers have to return to the hospital to get more of the drug to complete the treatment. And mothers may not always be willing or able to do that.

### 2. Access to comprehensive PMTCT service

**Figure 2:**
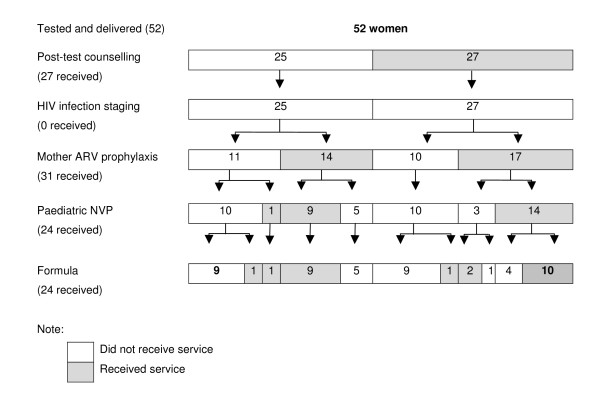
Use of comprehensive service for PMTCT among HIV positive women in Hanoi.

None of the women in the study received comprehensive PMTCT as recommended by WHO, because none were evaluated for their HIV infection stage. If we exclude HIV infection staging from the criteria of comprehensive PMTCT, still only 10 women and their children (<20%) received the remaining services (Figure 2). Moreover, there were nine women who did not receive any service at all. Among the other 33, although they did not benefit from all the recommended services, they did manage to access some.

One possible reason for under-use of PMTCT services is that women did not receive adequate counselling on PMTCT options. In this study, 22 women did not receive any counselling although they tested positive for HIV. Not only the quantity but also the quality of the counselling (as shown in Table 3) did not meet the required standards. The results revealed an emphasis in pre-test counselling on prevention of transmission of HIV, and not on what the test means, or what to do if it is positive. In the post-test counselling, again the emphasis was on disclosure and harm reduction, not on the needs of the women for care and protection. Even MTCT and how to prevent it only appeared in a small proportion of the interviews. Thirty-six women told us that they went to have another test at another facility in the hope of getting not only confirmation but also more information. And half of these women (18) went to at least one or two *more *testing centers before finally accepting the result and trying to find out what to do about it.

**Table 3:** Content of counselling on HIV testing

Content of counselling	Number of answers	Percentage
Content of pre-test counselling (N = 15)		
HIV infection risk	6	40.0
Explain about test result	3	20.0
Method to avoid HIV infection	10	66.7
Services available for HIV infected pregnant women	5	33.3

Content of post-test counselling (N = 27)		
Meaning of test result	5	18.5
Encourage to disclose test result	16	59.3
Plan for harm reduction	15	55.6
MTCT rate	13	48.1
Method to prevent MTCT	9	33.3
Family planning	5	18.5
Services available for HIV infected pregnant women	3	11.1

*"I visited different sites [3 testing sites] but only the institute X provided consultation, and referred me to Sunflower for further support. I think all clinics and VTC performing HIV tests need to provide the whole package of information on HIV including what can happen next and how to deal with it. It would be helpful to attract patients and to find a way for early treatment, with real support." *(28-year-old HIV infected mother)

The content of post-test counselling is limited. Women should receive counselling about breastfeeding and the associated risks of viral transmission. In Vietnam, breastfeeding is common and strongly promoted, so without counselling most women will expect to breastfeed.

*"When I nearly delivered, I went to hospital Y to check my blood and I found out that I was HIV positive. The doctor there did not counsel me anything so I still fed my baby by breast milk." *(28-year-old HIV infected mother, with HIV infected child)

One reason that women did not receive such counselling and other advice on care of themselves and their child is that people are only considered HIV positive when the confirmatory test is also positive. In the cases that the women had the first test at the time of labour, confirmation will follow only after some days. The health staff therefore would not provide counselling on ARV and formula until the test is confirmed and by then the woman may have left the hospital.

Figure 2 also shows clearly that women who received post-test counselling still did not always receive any other services. Some of these women explained that they considered HIV as a stigmatized disease and had bad experiences with health staff, and had therefore refused to deliver in the health facility:

*"I know that HIV is a dilemma, that's why I had very negative thinking. I thought that it would be the best if I did not deliver in this hospital. I delivered at my mother's home town"*. (27-year-old HIV infected mother, received no ARV prophylaxis)

Although free ARV combination prophylaxis is supposed to be available in Hanoi, among the 35 women being tested before 36 weeks of gestation who should have been able to receive that treatment, only 4 received it (Table 2). This result suggests that despite the availability of prophylactic ARV combination therapy, SD-NVP given at the time of delivery is still the standard prophylaxis in practice.

Even when women did get ARV combination prophylaxis, they often did not receive good explanations about how to use the drugs, or they were not used correctly by the health staff. Staff at all ARV treatment and prophylaxis sites was overworked and women complained about having to wait a long time to see staff. The four women who received prophylactic ARV combination starting from 36 weeks complained about the lack of counselling on adherence to the medicines. They did not know when they had to take the medicine and as a result, they may have had a different regime at home than in the hospital. In one case, the woman was already on ARV for her own health, but when she was admitted to hospital, the doctor gave her another dose for prophylaxis, without asking the woman first whether she was already on ARV. In another case, the pregnant woman was given ARV from two doctors in the same hospital, one who examined her during an ANC visit, and the other a relative who provided her with drugs for a different regimen. The women followed the advice of both doctors and as a result were taking a double dose of some drugs, most importantly Nevirapine, clearly an example of very poor patient management that put her life at risk. Two of these women said, about the barriers created by impractical administrative procedures:

*"I had to wait for the doctor to get medication from morning to afternoon, most of the time." *(24-year-old HIV infected mother)

*"I usually take medication at 7 am. But since I was hospitalized to wait for delivery, I take medication at 9 am because it's time for nurses to provide medication." *(26-year-old HIV infected mother)

### 3. Optional services

In the study, we explored experience of HIV-infected women in access to optional PMTCT services include abortion, which is legally available in Vietnam under standard conditions, and opting for Caesarean section, although the national guidelines do not consider HIV infection an indicator for that.

The time at which a woman is tested affects her choices. If women are tested early enough, or if they are already aware of their HIV status before becoming pregnant, they may want to opt for abortion. Table 1 shows that among the 50 women who found out their HIV status after getting pregnant, 40 were tested only after 22 weeks of gestation.

Moreover, counselling often lacked any advice about abortion for HIV infected women. One woman summarized this view:

*"One doctor was terrible. She told me to stand far from her. She asked me if I wanted to have an abortion. For an abortion, she would only give me if I would go home and get a letter with the signature of my parents. If I wanted to keep the child, it would be ok. So when I left, I wondered should I keep my baby or should I have an abortion? I wished at that time that the doctor could have given me advice and that we would have discussed the disease, the transmission rate from mother to infant, my financial situation, whether or not I could feed the child formula or what I would do if I died, who would take care of my child? But the doctor did not say anything." *(33 year-old- HIV infected woman)

When we asked the women were asked whether they had wanted to deliver by Caesarean section, 31 replied that they had wanted it, but only 9 women actually delivered that way. Many women expressed their idea that they could not have a Caesarean section because the health staff were afraid of HIV transmission:

*"I had pain for 4 days and I requested the doctors to give me an operation but they refused because they said if they would give me the operation, it would involve many people and they could all be infected so I had to deliver naturally." *(32-year-old HIV infected mother)

### 4. Stigma and discrimination: a cross cutting theme

Women experienced stigma and discrimination at all points of seeking services: counselling, ANC visits, abortion, delivery and post-delivery care. Many women revealed that they received poor care and did not want to revisit the hospital where they had delivered.

Among the 52 respondents, 14 reported that their test result was not kept confidential; most (10) received their results from the commune health station while the others were told through relatives.

*"They transferred the result from the hospital to the ward, from the ward to the sub-ward, she [the sub-ward leader] boomed out from the gate "Hey Q., T's wife, take HIV test for your son, you got it [HIV]". She made me so frustrated with her shouting. I was so ashamed. My husband told me I was very stupid, to give them our real address when I got tested, as if they were sending a gift [HIV test result] to us." *(29 year-old HIV infected mother)

Several women complained that they were not allowed to sit down during the counselling sessions, or that they had to cover the chair with newspaper before being allowed sitting down.

*"I was going to sit down but she [counsellor] said there was no need to sit." *(32-year-old HIV infected mother)

When the women were asked whether they had encountered any difficulties when they sought abortion, knowing their HIV positive status, 8 of 17 reported that they had. HIV infected women related negative experiences when seeking abortion:

*"My husband's aunty called a taxi scooter to take me to the private clinic for abortion because she said that if I go to hospital, they [health staff] will know my status and they will not do it for me." *(27-year-old HIV infected mother)

*"I found out I was HIV positive when I was 4 months pregnant. We [the couple] wanted to have an abortion but it is very hard. I went to many hospitals but the doctors refused me because I told them I was HIV positive. I had to tell them for them to prepare but they refused me. At the end, I went to hospital X. The doctor asked me to be hospitalized and gave me pills. After a week, I still could not abort. Then the doctor asked me to go home and come back after a week. Finally, I could abort with those pills." *(29-year-old HIV infected mother)

HIV infected women also described their experience of discrimination during delivery. Some women even tried to transfer to other cities for their delivery, to avoid the treatment they expected in the hospitals where they were known to be HIV positive. Of course, this solution is only available to women with sufficient financial means to make the transfer comfortable.

*"When they knew my HIV status, they shouted at me and did not allow me to sit, even when I was bleeding and was weak. They asked other patients to keep far away from me. Then they transferred me to a special room. When I gave birth, there was no staff, I gave normal birth, no operation." *(32-year-old HIV infected mother)

*"The doctors treated me well when they didn't know my status. But right after my delivery, they found that I was infected and they became rude. They did not tie the umbilical cord immediately. I was in so much pain." (*24-year-old HIV infected mother)

While follow-up care is a crucial component of comprehensive care and support for the HIV infected mother and her family after delivery, less than one fifth of the women were asked to come back to the hospital for an appointment.

Post-delivery care was also problematic. Nearly two thirds of the respondents reported that they had to stay in a separate room. About one third were not visited daily by health staff, nor did they have their temperature taken daily. HIV infected women reported feeling stigmatized because the care they and their children received was different from the care given to other women. Many women worried about not being able to keep their HIV status confidential when family, friends and other visitors could notice the difference in care and treatment.

*"I was in an isolated room when I woke up. Crying, my relatives stood far from me. I was not dressed and I left with only a thin sheet. Later on, I found out that the health staff informed all my relatives, neighbours, and friends who came to visit me of my status. I didn't understand why. Health care workers examined me carefully but said nothing. I couldn't see my beloved baby, either. Some days later, my husband told me everything; that I was infected with HIV." *(28-year-old HIV infected mother)

Data from in-depth interviews with HIV infected women showed that where the staff had had the benefit of training on caring for and working with HIV-infected patients, their attitudes could be much more positive. Clients of one hospital described the positive attitudes of the health staff there:

*"Doctors in hospital A are so nice. I thought they must have some negative attitude but they didn't. They did test for me and then move me to the infectious diseases department. Before doing tests, they also told me that they did HIV test. If I was infected, I would be moved to the infectious diseases department or if not, I would stay in normal department." *(37-year-old HIV infected mother)

## Discussion

**Figure 3:**
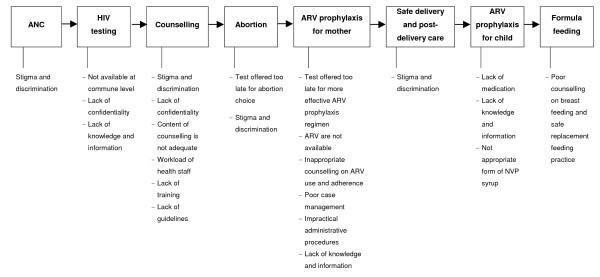
Accumulation of barriers to access PMTCT services for HIV positive women in Hanoi.

Worldwide, more than two million HIV-infected women give birth annually, but only 9% of them receive PMTCT intervention [21]. It is expected that having in place a simple PMTCT program that provides ARV prophylaxis for HIV infected mothers and children could increase the utilization of these services. However, our findings show that even in an urban area with sufficient resources, the PMTCT services were underused. The situation is similar in other developing countries [22,23]. The steps in the process to get adequate PMTCT and what could go wrong at each step are shown in Figure 3.

The study looked into the implementation of comprehensive PMTCT as recommended by WHO. In the context in which only about 44% of HIV positive pregnant women had access to minimal services, it is not surprising that only about 20% of them received the comprehensive service. While struggling to find their way to appropriate care among the fragmented services, the women experienced a number of problems including a high degree of felt stigma.

In one way, the fact that this study is about the extent to which 52 women, who were in a relatively advantageous position as members of a support group, and in a relatively well-serviced city, managed to get access to PMTCT can be considered a limitation. The results should not be considered as representative of the whole country. However, if the performance of the system shows so many gaps and weaknesses even in this advantaged setting, it could be expected that women with less support and knowledge and in more resource-limited settings elsewhere in Vietnam would receive even less adequate PMTCT. The results and recommendations would then apply even more to the needs of those women.

The HIV test is the entry point to getting HIV-infected women into a PMTCT program. The HIV test is routinely offered at health facilities and in Hanoi, the HIV testing uptake is quite high, 85%. The women in this study made many ANC visits, as did women in a broader scale study of women in Hanoi who had recently delivered [Thu Anh N, et al., submitted for publication]. Thus, with ARV available and in a supportive political environment, the minimal PMTCT intervention would be expected to be feasible. In contrast, we found that fewer than half of a group of 52 HIV-infected pregnant women in Hanoi had access to even minimal PMTCT. Other Asian nations have reported the same problem [23,24]. Reasons for unsatisfying performance in PMTCT programs included lack of HIV testing and/or of HIV testing early in pregnancy, poor quality counselling on possible PMTCT interventions, stock out of medication, and fragmentation of the health care system, especially weak referral systems which do not provide integrated case management between hospitals [11,25].

Counselling can play an important role in increasing access to PMTCT services. However, in general, counselling, including counselling on HIV/AIDS, is often not provided at the health facilities here [11,26]. Many of the HIV-infected women did not even receive any post-test counselling. That happened because they were not tested until delivery so there was no time to provide counselling, or because the health staff gave the test result to other people and lost the opportunity to provide counselling [11,27,28]. Even among those women who did receive counselling, the information provided was not sufficient to help them make decisions or cope with their problems [Thu Anh N, et al., submitted for publication]. In the context of the HIV epidemic, several guidelines on counselling have been developed by projects to improve the information provided to clients. Most of the guidelines, however, are adapted from the counselling guidelines for VCT sites which focus mainly on the high risk populations of drug users and sex workers, so that the counselling materials and training usually focuses on HIV prevention rather than on pregnancy or on care and support for HIV infected pregnant women [11,25].

Abortion is legally and socially accepted in Vietnam. Medical abortion is considered as an option among PMTCT interventions in many Asian countries [29-31]. However, many HIV infected pregnant women could not opt for abortion because they were offered HIV testing too late in their pregnancy. Even if they were tested early enough, some reported difficulties in accessing abortion services if they disclosed their HIV status to the health staff. A weak point is that HIV, abortion and family planning counselling services are not integrated; health care workers suspected a large loss of follow up although no numbers were available [25,23].

Compared to ARV for treatment, the administration of ARV as prophylaxis for PMTCT is much simpler [23]. However, observation at PMTCT sites revealed that HIV-infected women did not receive appropriate counselling on use of ARV. There were also very poor patient management and impractical administration procedures.

Breastfeeding is highly socially desirable in Vietnam as in other Asian countries, but the practice of exclusive breastfeeding is very limited [26,32]. In Vietnam, replacement feeding is routinely recommended for HIV infected mothers. However, the study found that social and cultural barriers confront HIV infected women who they decide not to breastfeed their child. Among the women who decided to not breastfeed, because the counselling they received was inadequate, many of them did not receive instructions on safe preparation of formula, which may lead to high risk of diarrhoea and other diseases for the newborn [11]. There is little data as yet to support the effectiveness and safety of replacement feeding in the context of Vietnam's culturally determined infant feeding patterns and climate, and the financial means of HIV infected women. Global evidence suggests that women are put under extreme pressure to adhere to traditional feeding patterns if they have not been able to disclose their HIV status at home [10].

Many women told us that fear of stigma and discrimination was the most important barrier for them to use HIV testing services [33]. As the epidemic in Vietnam is still concentrated among drug users and sex workers, HIV infection has been associated with "social evils" and "immoral behaviour" [34]. An HIV test is not simply about information; it involves social relationships and strong emotions. Most HIV-infected people are fearful of the result and of other people knowing their status and believe that if they are found to be positive, their test result will not remain secret [8,35]. The official notification system follows a public health approach, which has been applied to control infectious diseases in Vietnam for long time. In that system, the positive HIV test results are shared with health staff at district and commune levels, supposedly to ensure care for the HIV-positive person in the community. In the cases when pregnant women were tested only when they came to the hospital already in labour, their test results were shared with their relatives, without asking for consent. To keep their test results confidential, women who suspect their status and know how the system works often provided false names and addresses to avoid the official notification system [35].

Many infected women expressed their dissatisfaction in the way that some counsellors treated them. Inappropriate communication about HIV status can result in the women's avoiding the health services, which means they will not later be able to access the continuous treatment, care and support they need. The quality of post-delivery care is believed to influence the reproductive health outcome and use of health services after birth. A survey in seven provinces in Vietnam revealed that the knowledge of health staff on routine post-delivery care was quite sufficient and the quality of routine reproductive services at district and commune health station was good [15]. Nevertheless, too careful and not always kind attention was paid to HIV-infected women during post-delivery care, which led to perceived stigma and discrimination among those receiving care.

In the context of the high HIV testing uptake in Hanoi, the findings suggest feasible interventions to increase the use of PMTCT service. A number of studies have demonstrated that lack of training and lack of time are the main factors affecting the quality of counselling. Also, the negative attitudes of health staff towards HIV infected persons may prevent them having access to health care [7,11,33,34]. That suggests that the quality of counselling on PMTCT could be enhanced by improving capacity of the counsellors and by making PMTCT guidelines available, including counselling guidelines appropriate to high- and low-workload facilities and including culturally appropriate infant feeding advice. A positive atmosphere in the ANC facilities should be promoted by normalizing HIV related services and undertaking behaviour change communication campaigns aimed at the health facilities. Feedback from service users should be used as one way to evaluate the quality of service.

On the other hand, women who were notified through the official system of their HIV positive status reported the lack of support from family, social isolation and poor care in health facilities [35]. The results of the study suggest that it would be better to make HIV testing anonymous for pregnant women and allowing HIV positive pregnant women choice in disclosure routes as well as where to use other services. In a country like Vietnam, with high ANC coverage, it should be possible to offer HIV testing in the first trimester to increase women's choices in PMTCT. The most recent guidelines produced for PMTCT in Vietnam have included this recommendation, at least for areas with a high number of mothers at risk, partly on the basis of the results of this research.

Finally, the health facilities should not only make ARV available but also develop a client-friendly approach to distribute medication with adequate counselling on its use and adherence, to fulfil the basic requirements for good patient management.

These results not only point the way to improvements in provision of PMTCT in Vietnam but may also contribute to the picture of PMTCT in low-prevalence countries, especially in Southeast Asia, which may share features with that in the better-described systems in sub-Saharan Africa but in other ways may be different, and may need different investments to provide needed services.

## Competing interests

The authors declare that they have no competing interests.

## Authors' contributions

TAN participated in the design of the study, conduct the data collection and the dada analysis. PO participated in the design of the study and carried out interviews. YPN carried out the data collection and the data analysis. PW conceived of the study and participated in its design and coordination. AH participated in the study design, the data analysis, and coordination. All authors read and approved the final manuscript.
